# Supplementary material for: Metabolome and Transcriptome Profiling Reveal That Four Terpenoid Hormones Dominate the Growth and Development of Sanghuangporus baumii
Source: J Fungi (Basel). 2022 Jun 21;8(7):648. doi: 10.3390/jof8070648 (PMC9317858; doi:10.3390/jof8070648)
Supplement: Supplementary file 1 [file jof-08-00648-s001.zip › jof-1780801-supplementary.pdf]

Supplementary Data

Metabolome and transcriptome profiling reveals that terpenoid biosynthesis dominates the growth and development of *Sanghuangporus baumii*

Zengcai Liu[1], Xinyu Tong[1], Ruipeng Liu[1], Li Zou[1,*]

[1]College of Forestry, Northeast Forestry University, Harbin 150040, China;

1758458181@nefu.edu.cn (Z.L.); hhxxyx@nefu.edu.cn (X.T.);

liuruipeng@nefu.edu.cn (R.L.); shyj@nefu.edu.cn (L.Z.)

* Corresponding author: shyj@nefu.edu.cn; Tel.: +86-0451-86660457

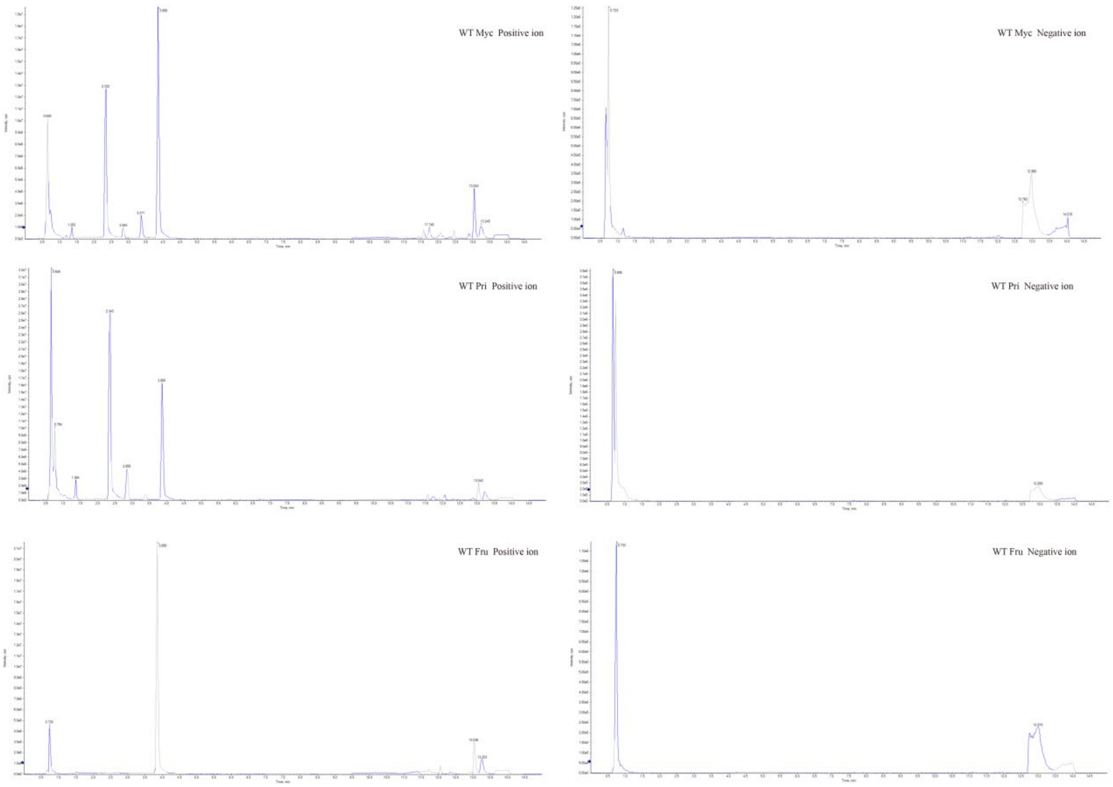

Figure S1. The base peak chromatogram of metabolites.

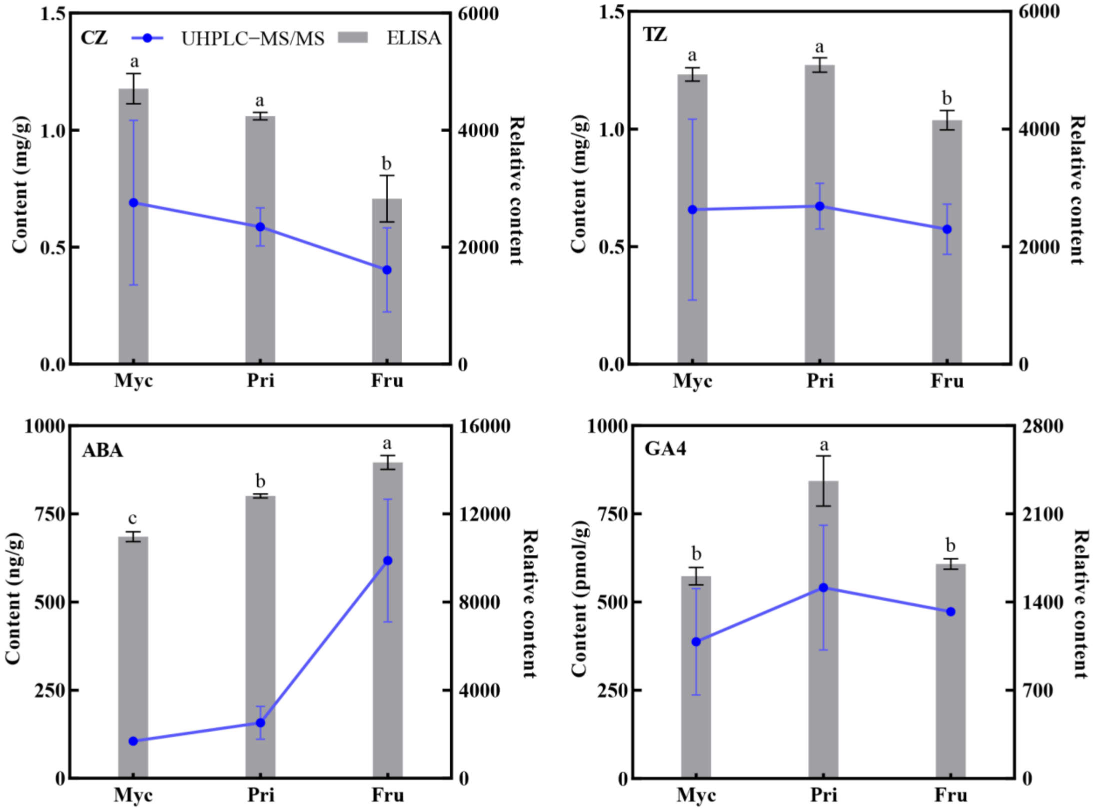

Figure S2. Results of ELISA and UHPLC-MS/MS determination of four terpenoid hormones.

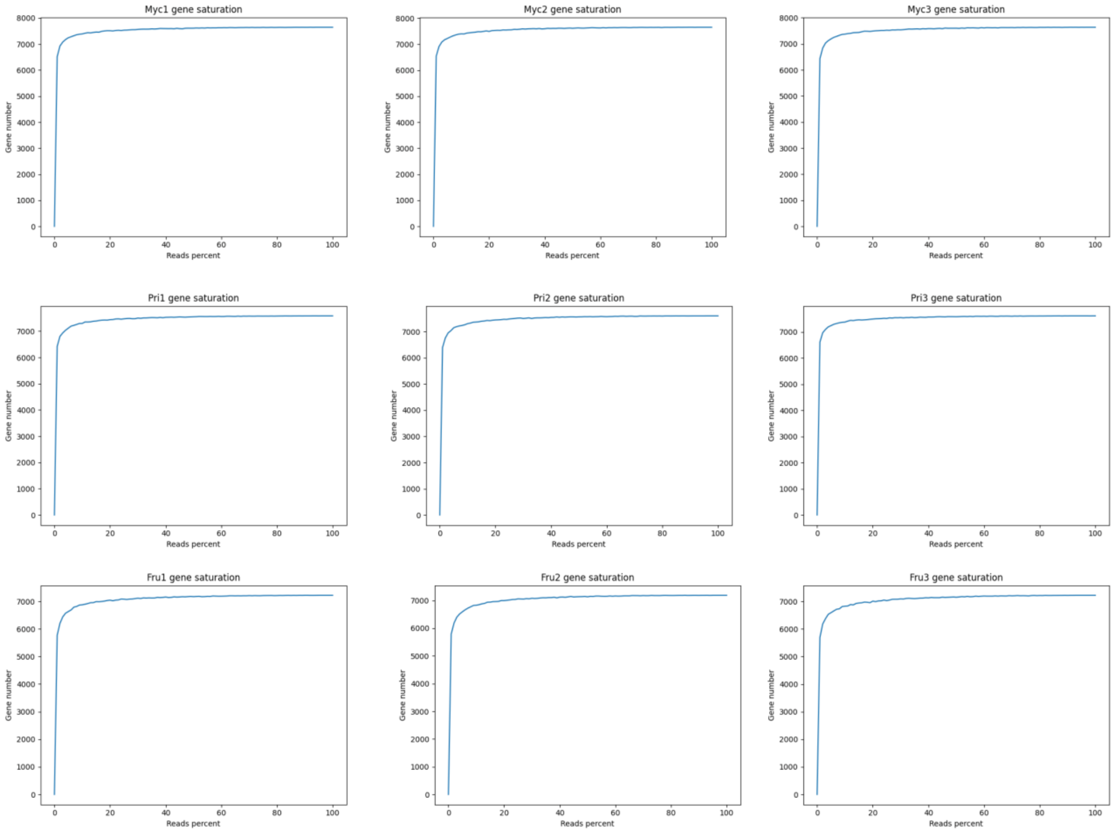

Figure S3. Sequencing saturation of nine samples.

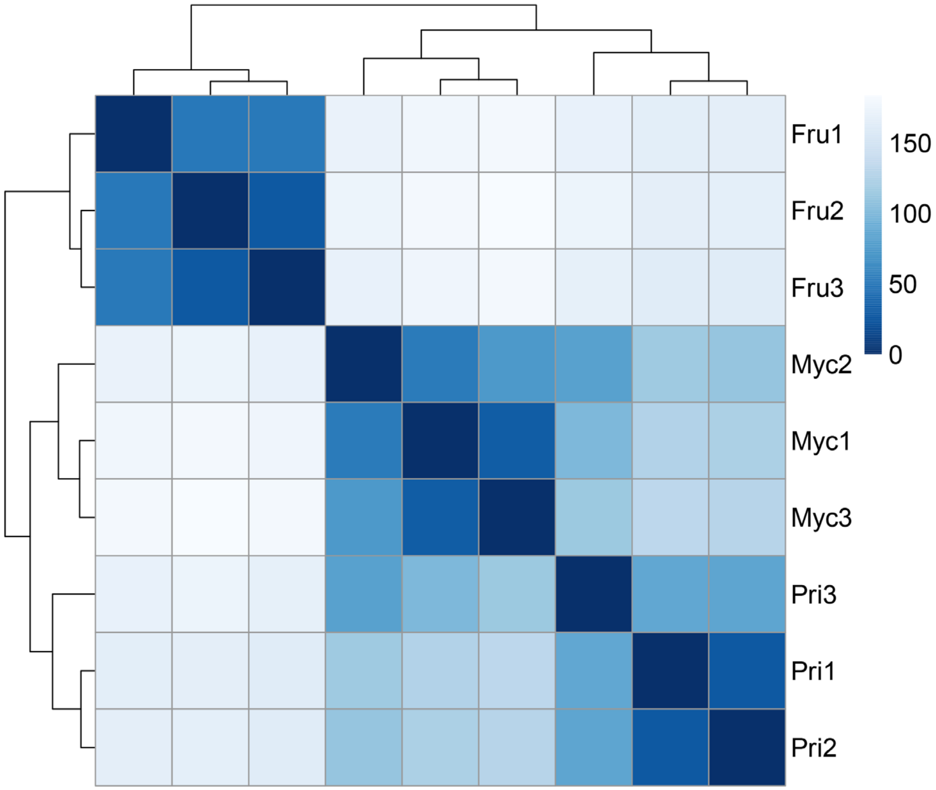

Figure S4. Gene expression patterns in the three development stages.

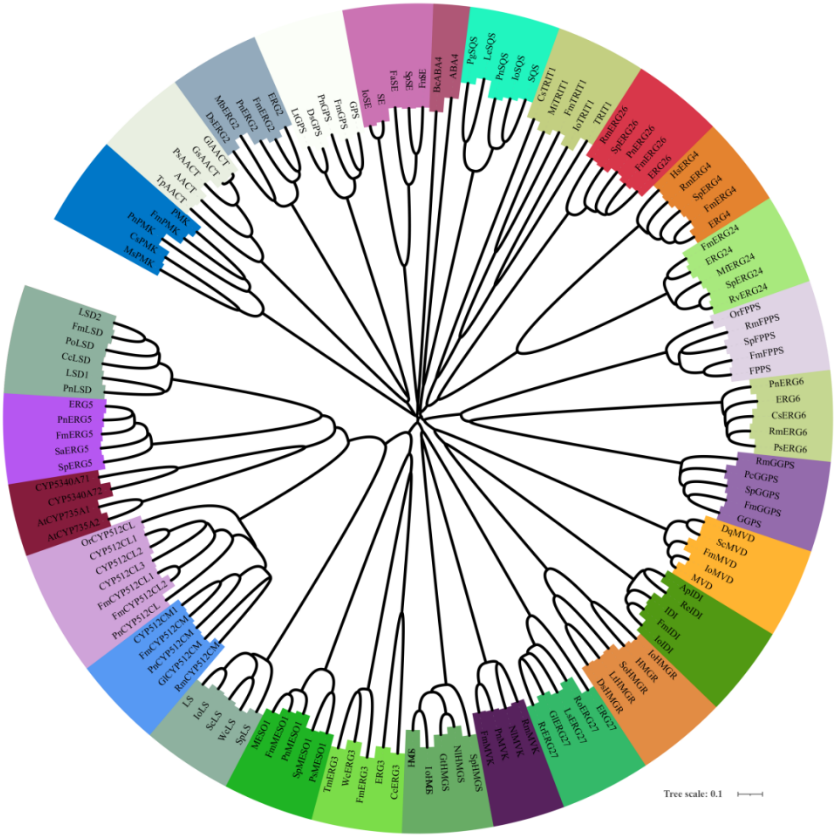

Figure S5. Phylogenetic analysis of terpenoid biosynthesis genes of *S. baumii*.

>OCB87509.1 AACT [Sanghuangporus baumii]; >AEB00648.1 GlAACT [Ganoderma lucidum];>OJT13330.1 TpAACT [Trametes pubescens]; >GJE84702.1 PsAACT [Phanerochaete sordida];>PIL36744.1 GsAACT [Ganoderma sinense]

>OCB86311.1 HMGS [Sanghuangporus baumii]; >QEP49708.1 IoHMGS [Inonotus obliquus]; >XP_007865030.1 GtHMGS [Gloeophyllum trabeum]; >KZT21597.1 NlHMGS [Neolentinus lepideus]; >KLO11524.1 SpHMGS [Schizopora paradoxa]

>OCB83928.1 HMGR [Sanghuangporus baumii]; >AEX09818.1 IoHMGR [Inonotus obliquus]; >TCD61858.1 SoHMGR [Steccherinum ochraceum]; >XP_007363597.1 DsHMGR [Dichomitus squalens]; >RPD57004.1 LtHMGR [Lentinus tigrinus]

>OCB84761.1 MVK [Sanghuangporus baumii]; >XP_007263021.1 FmMVK [Fomitiporia mediterranea]; >PAV16450.1 PnMVK [Pyrrhoderma noxium]; >KZT29708.1 NlMVK [Neolentinus lepideus]; >TDL25608.1 RmMVK [Rickenella mellea]

>OCB89752.1 PMK [Sanghuangporus baumii]; >XP_007267080.1 FmPMK [Fomitiporia mediterranea]; >PAV19036.1 PnPMK [Pyrrhoderma noxium]; >KAF9012325.1 CsPMK [Cyathus striatus]; >KAF7376157.1 MsPMK [Mycena sanguinolenta];

>OCB90355.1 MVD [Sanghuangporus baumii]; >XP_007266924.1 FmMVD [Fomitiporia mediterranea]; >QEP49711.1 IoMVD [Inonotus obliquus]; >XP_027611478.1 ScMVD [Sparassis crispa]; >KZT71761.1 DqMVD [Daedalea quercina]

>OCB86091.1 IDI [Sanghuangporus baumii]; >QEP49712.1 IoIDI [Inonotus obliquus]; >XP_007265734.1 FmIDI [Fomitiporia mediterranea]; >KAF8506685.1 ReIDI [Russula emetica]; >KAF9568402.1 ApIDI [Agrocybe pediades]

>OCB89018.1 GPS [Sanghuangporus baumii]; >XP_007271176.1FmGPS [Fomitiporia mediterranea]; >PAV22679.1 PnGPS [Pyrrhoderma noxium]; >XP_007360550.1 DsGPS [Dichomitus squalens] >RPD65057.1 LtGPS [Lentinus tigrinus]

>OCB92206.1 FPPS [Sanghuangporus baumii]; >XP_007270730.1 FmFPPS [Fomitiporia mediterranea]; >KLO15136.1 SpFPPS [Schizopora paradoxa]; >TDL20808.1 RmFPPS [Rickenella mellea]; >OCH84930.1 OrFPPS [Obba rivulosa]

>OCB88125.1 GGPS [Sanghuangporus baumii]; >XP_007262248.1 FmGGPS [Fomitiporia mediterranea]; >KLO15127.1 SpGGPS [Schizopora paradoxa]; >TDL22985.1 RmGGPS [Rickenella mellea]; >KAF9483315.1 PcGGPS [Pholiota conissans]

>OCB88085.1 TRIT1 [Sanghuangporus baumii]; >AXF50742.1 IoTRIT1 [Inonotus obliquus]; >XP_007267488.1 FmTRIT1 [Fomitiporia mediterranea]; >KAF9001991.1 CsTRIT1 [Cyathus striatus]; >XP_037216540.1 MiTRIT1 [Mycena indigotica]

>OCB91754.1 CYP5340A71 [Sanghuangporus baumii]; >OCB92139.1 CYP5340A72 [Sanghuangporus baumii]; >OAO90393.1 AtCYP735A1 [Sanghuangporus baumii]; >OAP14242.1 AtCYP735A2 [Sanghuangporus baumii]

>OCB90839.1 ABA4 [Sanghuangporus baumii]; >XP_001553969.2 BcABA4 [Botrytis cinerea]

>OCB86527.1 CYP512CL1 [Sanghuangporus baumii]; >OCB86532.1 CYP512CL2 [Sanghuangporus baumii]; >OCB86528.1 CYP512CL3 [Sanghuangporus baumii]; >XP_007261441.1 FmCYP512CL1 [Fomitiporia mediterranea]; >XP_007261418.1 FmCYP512CL2 [Fomitiporia mediterranea]; >PAV19648.1 PnCYP512CL [Pyrrhoderma noxium]; >OCH95708.1 OrCYP512CL [Obba rivulosa]

>OCB90766.1 CYP512CM1 [Sanghuangporus baumii]; >XP_007261732.1 FmCYP512CM [Fomitiporia mediterranea]; >PAV15339.1 PnCYP512CM [Pyrrhoderma noxium]; >KAF9225192.1 GlCYP512CM [Gyrodon lividus]; >TDL22831.1 RmCYP512CM [Rickenella mellea]

>OCB89274.1 SQS [Sanghuangporus baumii]; >AEX09819.1 IoSQS [Inonotus obliquus]; >PAV19342.1 PnSQS [Pyrrhoderma noxium]; >GAW09328.1 LeSQS [Lentinula edodes]; >TFL01662.1 PgSQS [Pterula gracilis]

>OCB87673.1 SE [Sanghuangporus baumii]; >QEP49718.1 IoSE [Inonotus obliquus]; >XP_007269752.1 FmSE [Fomitiporia mediterranea]; >KLO10978.1 SpSE [Schizopora paradoxa]; >KAF8959626.1 FaSE [Flammula alnicola]

>OCB89993.1 LS [Sanghuangporus baumii]; >QEP49720.1 IoLS [Inonotus obliquus]; >KLO19286.1 SpLS [Schizopora paradoxa]; >XP_027617410.1 ScLS [Sparassis crispa]; >PCH44389.1 WcLS [Wolfiporia cocos]

>OCB87224.1 LSD1 [Sanghuangporus baumii]; >OCB90054.1 LSD2 [Sanghuangporus baumii]; >PAV21443.1 PnLSD [Pyrrhoderma noxium]; >XP_007263187.1 FmLSD [Fomitiporia mediterranea]; >XP_001836522.1 CcLSD [Coprinopsis cinerea]; >XP_036628375.1 PoLSD [Pleurotus ostreatus]

>OCB87985.1 ERG24 [Sanghuangporus baumii]; >XP_007264907.1 FmERG24 [Fomitiporia mediterranea]; >OAX40973.1 RvERG24 [Rhizopogon vinicolor]; >XP_041171058.1 SpERG24 [Suillus paluster]

>KAF9267816.1 MfERG24 [Marasmius fiardii]

>OCB84697.1 MESO1 [Sanghuangporus baumii]; >XP_007262853.1 FmMESO1 [Fomitiporia mediterranea]; >PAV16076.1 PnMESO1 [Pyrrhoderma noxium]; >KLO07398.1 SpMESO1 [Schizopora paradoxa];>XP_007388216.1 PsMESO1 [Punctularia strigosozonata]

>OCB87751.1 ERG26 [Sanghuangporus baumii]; >XP_007270754.1 FmERG26 [Fomitiporia mediterranea]; >KLO07614.1 SpERG26 [Schizopora paradoxa]; >PAV17340.1 PnERG26 [Pyrrhoderma noxium]

>TDL22249.1 RmERG26 [Rickenella mellea]

>OCB86242.1 ERG27 [Sanghuangporus baumii]; >KAF8583973.1 RrERG27 [Ramaria rubella]; >XP_040770639.1 LsERG27 [Laetiporus sulphureus]; >KAF9224357.1 GlERG27 [Gyrodon lividus]; >KAF8485315.1 RoERG27 [Russula ochroleuca]

>OCB86578.1 ERG6 [Sanghuangporus baumii]; >KAF9010079.1 CsERG6 [Cyathus striatus]; >PAV15143.1 PnERG6 [Pyrrhoderma noxium]; >XP_007384312.1 PsERG6 [Punctularia strigosozonata]; >TDL18780.1 RmERG6 [Rickenella mellea]

>OCB90543.1 ERG2 [Sanghuangporus baumii]; >XP_007270358.1 FmERG2 [Fomitiporia mediterranea]; >PAV15490.1 PnERG2 [Pyrrhoderma noxium]; >KAF9234931.1 MbERG2 [Melanogaster broomeanus];>XP_007366653.1 DsERG2 [Dichomitus squalens]

>OCB89410.1 ERG3 [Sanghuangporus baumii]; >KAG2017788.1 CcERG3 [Coprinopsis cinerea]; >XP_007263262.1 FmERG3 [Fomitiporia mediterranea]; >KAF8235768.1 TmERG3 [Tricholoma matsutake]; >PCH42137.1 WcERG3 [Wolfiporia cocos]

>OCB85193.1 ERG3 [Sanghuangporus baumii]; >XP_007265848.1 ERG3 [Fomitiporia mediterranea]; >PAV22239.1 ERG3 [Pyrrhoderma noxium]; >KLO20001.1 ERG3 [Schizopora paradoxa] >KAG2045300.1 ERG3 [Suillus americanus]

>OCB87800.1 ERG4 [Sanghuangporus baumii]; >XP_007261639.1 FmERG4 [Fomitiporia mediterranea]; >KLO12723.1 SpERG4 [Schizopora paradoxa]; >TDL23613.1 RmERG4 [Rickenella mellea]; >TFK53410.1 HsERG4 [Heliocybe sulcata]

Table S1. Primers for qRT-PCR analysis.

Gene ID	Primers	Sequences 5´→3´
gene-A7U60_g6623	HMGS-F	CGAGAAGACTGCGAGGCGATAC
	HMGS-R	TGGGACTTGAGGGATTGGAGAGG
gene-A7U60_g9137	HMGR-F	TGGCTTTGAACGCCGTCTTA
	HMGR-R	CTCTCCATTTCGTCGCTATCACC
gene-A7U60_g2454	MVD-F	TTTCGTATTTCCCGCAAGCCCAG
	MVD-R	GCATTCAGCAAAGCCTCCTCAGC
gene-A7U60_g6678	IDI-F	GCTGCTGCGGCGTTTGCTACTAT
	IDI-R	ATCTTCTCGGATGCTCGTTGCTG
gene-A7U60_g4872	TRIT1-F	GGAAGGTCCTCCGTAATCTTGAAA
	TRIT1-R	AAGCGACTGTCCGCAGCCGTG
gene-A7U60_g982	CYP5340A71-F	GTCTTACTTGTGGCGGGGTATGAAA
	CYP5340A71-R	ATAGGTTGGGTCCCTGTTAGAAAAT
gene-A7U60_g3826	GPS-F	CCCCTACTCCTTCTCATCCATCAA
	GPS-R	GCCGCACCATCTATCACATCATCA
gene-A7U60_g1950	ABA4-F	ACAGCAGACGGGAAGTTCTACAAA
	ABA4-R	CTACTGCTCTACCGATGCCACTGT
gene-A7U60_g402	FPPS-F	CCGTGGCATTTCCGTAACAGAC
	FPPS-R	CAGCAGGGTTGTCCTCTCCGTGT
gene-A7U60_g4753	GGPS-F	ATGGTAAATGATAAAACTGGTGGTC
	GGPS-R	TTGCTCTGAAGATTGAAATAGTCGT
gene-A7U60_g6423	CYP512CL1-F	TTCACACACGCCTTGTTCCACTTAG
	CYP512CL1-R	CGTTCATTCGCTGGCTTTCTTTCA
gene-A7U60_g3573	SQS-F	TCCTGCTGCTCATCACACATCCTC
	SQS-R	CCAATAAGTGCCAACATCGCTTCA
gene-A7U60_g5199	SE-F	TAAGAACTCGGGGACTGAAACGAAG
	SE-R	CTTGAGAATCGCACCGACGAAGTAG
gene-A7U60_g2849	LS-F	ACACAGTTCGCCCTTGAGAGCC
	LS-R	CATCTTCACGGCCCGTTCGATAGGT
gene-A7U60_g5741	LSD1-F	CATTGGCTTCCTATTATTGGCTCC
	LSD1-R	GGGTCGTGAAGTGACAATAAGCAT
gene-A7U60_g5073	ERG26-F	ACAACAAACAAACGCACTTCCAGC
	ERG26-R	ACACGACGAACACCCGTTGAGAGA
	α-tubulin-F	CCAGCAAGCGTTACCGATT
	α-tubulin-R	TCCACGACGTCCATCGTTC

Table S2. Detected terpenoid metabolites using widely targeted metabolome analyses in *S. baumii*.

Compounds Name	CAS	KEGG_ID	Formula	EXACT_MASS	q1	q3	ionmode	tention ti	mMyc1	mMyc2	mMyc3	mPri1	mPri2	mPri3	mFru1	mFru2	mFru3
cis-Zeatin	32771-64	C15545	C10H13N5O	219.112	220	136	+	3.6	1043.51	2746.34	4496.17	1932.75	2729.55	2378.01	2538.68	1510.04	783.406
trans-Zeatin	1637-39-	C00371	C10H13N5O	219.112	220	136	+	3.41	4290.886	1476.14	766.23	3548.94	2773.33	2924.18	2898.97	1923.15	2072.43
(1R)-(-)-Menthyl a	2623-23-	C09870	C12H22O2	198.16198	199.2	157.2	+	10.4896	2676.397999	5322.99	6521.72	8979.62	11122.5	2328.5	11434.2	4880.61	8782.5
2,6-Dimethyl-7-oct	73815-21	-	C10H20O3	188.141245	189.1	153.1	+	3.81923	2090.377	1342.65	3577.75	4040.21	2205.35	1958.54	4437.54	2477.93	2532.42
Bornyl acetate	5655-61-	C09837	C12H20O2	196.14633	197.2	137.1	+	6.54107	2046.667	4733.35	1655.81	7947.95	3378.97	11572.7	6230.3	4192.13	8128.07
Cantharidin	56-25-7	C16778	C10H12O4	196.07356	197.1	95.1	+	6.0265	12312.6725	2407.75	6387.58	5361.25	5143.84	12961.9	8594.33	10559.8	11032.8
Carvacryl acetate	6380-28-	-	C12H16O2	192.11503	193.1	133.1	+	11.228	16310.433	10257.4	13412.2	14863.7	17038.1	4382.3	13376.6	14015	12105.7
Citronellyl acetate	141-11-7	C12298	C12H22O2	198.16198	199.2	55.1	+	10.4819	8030.282998	11387.9	5232.13	5739.2	8022.72	12756.9	5872.24	9087	4234.64
Isopulegol	7786-67-6;89-79-2		C10H18O	154.135765	155.1	137.1	+	2.46032	34932.775	30176.4	26908.3	10790.5	5163.16	5399.65	5839.51	5558.77	3554.93
Nerylacetate	141-12-8	-	C12H20O2	196.14633	219.1	81.1	+	8.37492	7464.543	4327.62	5929.25	11315.6	7117.6	14434.1	10838.9	16975.1	5866.27
p-Cymene	99-87-6	C06575	C10H14	134.10955	135.1	93.1	+	11.6207	39572.09649	25754.2	24573.3	29834.2	13039.7	25883.6	12338	41511.7	13133.9
Picrocrocin	138-55-6	C17055	C16H26O7	330.167855	331.2	151.1	+	9.24762	774.1695	1248.31	2716.96	9805.08	4498.09	2584.35	1040.36	3154.36	1277.27
Terpinine-4-ol	562-74-3	C17073	C10H18O	154.135765	154.1	43	+	5.45468	8779.376	10991.3	9731.99	9568.57	4916.34	28781.4	14314.8	13256.2	10932.4
Terpinolene	586-62-9	C06075	C10H16	136.1252	136.1	93.1	+	10.5735	23040.96749	23261.6	24260	13089.9	18515.4	17993.6	14307.4	29323.9	12577.9
Abscisic acid	21293-29	C06082;C11	C15H20O4	264.13616	265.1	247.1	+	7.86	1449.756	1931.74	1669.8	1525.28	2728.2	3321.85	11957.7	5926.4	11749.3
Alantolactone	546-43-0	C09289	C15H20O2	232.14633	233.2	105.1	+	11.9329	25788.6805	23531.9	28362.8	23690.6	23737.9	26668.4	41493.9	64414.7	46416.5
Alpha-caryophylle	6753-98-	C09684	C15H24	204.1878	205.2	81.1	+	11.581	26155.0205	43268.8	31565.3	21268.3	27453.8	11692.8	34163.5	38182.8	45286
alpha-Cyperone	473-08-5	C17090	C15H22O	218.167065	219.2	67.1	+	12.2018	86135.845	42215.4	36972.9	47311.6	48285.1	36013.3	294582	130183	254447
Alpha-Santonin	481-06-1	C02206	C15H18O3	246.125595	247.1	173.1	+	7.86777	12563.7855	7283.96	8550.43	15421.5	10288.4	11216.8	8337.57	17488.3	14756.4
Anisatin	5230-87-	C09294	C15H20O8	328.11582	329.1	311.1	+	5.42288	-	7657.68	2118.35	3766.23		6791.81	10190.1	2229	3981.14
Artemisinin	63968-64	C09538	C15H22O5	282.146725	283.2	110.1	+	13.0238	204397.6715	167578	181010	254880	292914	168293	171513	133280	149300
beta-Costic acid	3650-43-	-	C15H22O2	234.16198	235.2	189.2	+	8.89518	4539.213	7108.36	4750.23	3675.88	9631.33	12161.4	2048.59	7545.86	4815.52
Bisabolol oxide A	22567-36	C16773	C15H26O2	238.19328	239.2	55.1	+	11.4953	75627.36051	46864.9	78679.7	80813.2	50083.3	54492.1	62189.6	82787.5	59393.1
Catalpalactone	1585-68-	C16929	C15H14O4	258.08921	259.1	67.1	+	12.8392	2596.440001	6210.18	8500.73	2559.68	3752.99	2984.43	4185.92	7291.48	5155.83
Costunolide	553-21-9	C09382	C15H20O2	232.14633	233.2	161.1	+	12.2077	5718.183	16060.6	15376.5	2459	8707.04	13583.6	19367	7139.22	7948.59
Cryptomeridiol	4666-84-	C17676	C15H28O2	240.20893	241.2	223.2	+	3.23935	1205.2035	2928.34	1418.9	2955.78	4879.17	4489.31	5113.8	746.045	1787.21
Curzerene	17910-09	-	C15H20O	216.151415	217.2	55.1	+	2.54623	6776.381	7016.13	34304.9	7758.25	17814	13741.8	13188.7	7169.92	5948.53
Epitulipinolide	24164-13	C09566	C17H22O4	290.15181	291.2	231.1	+	5.06868	2823.0645	595.404		5461.84	3075.61	5037.96	2427.39	1846	3997.1
Eupatolide	6750-25-	C09440	C15H20O3	248.141245	249.1	231.1	+	7.17532	486.588	1485.54	4759.4	1600.63	1569.43	1657.84	4848.07	2335.49	1034.45
Isoalantolactone	470-17-7	C09484	C15H20O2	232.14633	233.2	105.1	+	12.3094	20204.9525	17258.4	8250.19	11871.2	14077.6	10610.5	13097.8	11925.1	26269.5
Lactupicrin	65725-11	C09490	C23H22O7	410.136555	411.1	259.1	+	13.7958	2315.868499	1930.41	3649.37	9938.35	5408.67	994.663	4823.8	2566.12	2112.93
Linderalactone	728-61-0	C17425	C15H16O3	244.109945	245.1	199.1	+	8.73737	3744.7345	5662.77	7828.22	4082.97	1110.56	4371.48	3222	2542.66	1713.66
Pterosin D	34169-70	-	C15H20O3	248.141245	249.1	231.1	+	3.2902	3091.383	10803.7	1635.58	2963.07	717.111	7917.4	3556.58	2955.47	2532.16
Pyrocurzerenone	20013-75	-	C15H16O	212.120115	213.1	171.1	+	3.42397	655.752	1467.13	2468.2	633.264	1921.76	1963.76	1669.15	3340.8	1153.35
Tenulin	19202-92	C09557	C17H22O5	306.146725	307.2	289.1	+	4.22953	1177.922	3081.92	2411.8	934.032	957.386	8437.58	4144.54	2561.95	2511.58
trans-Caryophyller	87-44-5	C09629	C15H24	204.1878	205.2	95.1	+	12.1185	29115.75499	35357.4	31663.5	21225.8	43785.9	13016	134669	58124.7	118807
Valerenic acid	3569-10-	C09743	C15H22O2	234.16198	235.2	161.1	+	11.2448	19685.6415	3997	20300	15722.9	10903.5	10100.9	14684.8	22095.9	29091.3
Gibberellin A4	468-44-0	C11864	C19H24O5	332.1624	331	243	-	9.98	881.699	702.217	1672.71	1082.03	2209.49	1256.15	1299.2	1353.74	1322.1
(+)-Glaucarubinone	1259-86-	C08763	C25H34O10	494.2152	495.2	477.2	+	5.79035	1729.242	3534.51		2328.96	5286.77	13173.9	6980.2	3760.12	2805.24
6,7-Dehydroferrugi	34539-84	-	C20H28O	284.214015	285.2	269.2	+	9.06125	11835.9695	5507.89	17128.3	19267.7	15131.1	9865.96	9157.35	8221.14	8205.82
Abietic acid	514-10-3	C06087	C20H30O2	302.22458	303.2	285.2	+	5.20292	5654.0975	17453.1	18707.1	6074.63	4638.37	13231.7	5416.34	5376	4790.33
Ailanthone	981-15-7	C08747	C20H24O7	376.152205	377.2	359.1	+	3.68698	2728.745	1799.33	1960.29	1679.13	4377.9	1806.6	924.903	1483.68	2920.07
Bruceine D	21499-66	C08752	C20H26O9	410.157685	411.2	393.2	+	4.83543	3284.301	960.918	508.407	5749.07	2782.2	36767.8	10673.7	9948.8	15703.9
Cafestol	469-83-0	C09066	C20H28O3	316.203845	317.2	281.2	+	5.47147	1567.223	2060.46		1032.46	538.16	8957.09	2166.7	4291.9	1039.43
Carnosol	5957-80-	C09069	C20H26O4	330.18311	329.2	285.2	-	12.147	1161.526001	751.237		1567.26	1400.17	2427.42	1322.15	1805.74	1336.47
Diosbulbin D	66756-57	-	C19H20O6	344.12599	345.1	327.1	+	2.6289	1015.56	2048.42	3430.96	2790.65	2282.96	7277.72	3118.94	4927.36	2950.51
ent-16beta,17-dihy	55483-24	-	C20H30O4	334.21441	335.2	271.2	+	7.14977	1136.1025	1029.89	761.176	1348.54	2056.98	7097.24	4364.34	2249.56	6468.05
Eurycomalactone	23062-24	C08759	C19H24O6	348.15729	349.2	331.2	+	5.79035	2185.0175	2263.12	2855.74	5480.83	5992.9	12167.7	5790.83	7180.84	1369.04
Goshonoside F1	90851-24	-	C26H44O8	484.30362	485.3	449.3	+	12.3021	-	-	11098.1	14485.8	7230.62	6047.41	5421.55		4757
Kaurenoic acid	6730-83-	C11874	C20H30O2	302.22458	303.2	257.2	+	5.90232	-	1824.87		1408.81	79448.8	54079.2	4762.61	6202.2	3412.04
Kirenol	52659-56	-	C20H34O4	338.24571	321.2	81.1	+	13.158	236324.4471	298191	302235	317905	336970	293424	260835	341036	272756
Lathyrol	34420-19	C09125	C20H30O4	334.21441	335.2	317.2	+	7.14977	1000.818	1545.84	1760.98	3491.46	6299.73	8814.79	6550.61	5850.19	4959.81
Methyl tanshinona	18887-19	-	C20H18O5	338.115425	339.1	171.1	+	11.4294	5627.605501	2859.95	13537.9	6721.5	7007.97		5626.75	2042	2914.39
Mevastatin	73573-88	C13963	C23H34O5	390.240625	391.2	185.1	+	12.0672	8410.5	25311.9	26475.7	22654.7	28872	26733.7	15315.4	22775.5	13767.1
Miltirone	27210-57	C13715	C19H22O2	282.16198	283.2	55.1	+	12.9769	1253881.584	1626497	1456219	2042345	2009322	1196884	947023	1339745	1149932
Sterebin A	107647-1	-	C18H30O4	310.21441	311.2	293.2	+	11.7548	243260.6951	524795	1065600	122048	169697	85995.4	60845.5	45643.5	32822.5
Sterebin E	114343-7	-	C20H34O4	338.24571	339.3	303.2	+	13.1931	5772.096001	3763.07	8685.31	7484.95	114946	8911.68	3394.33	3281.9	4873.98
Tripdiolide	38647-10	C09202	C20H24O7	376.152205	377.2	359.1	+	5.13582	2402.7325	1190.6	5898.8	9167.17	6913.23	11959.5	4667.51	7720	4268.98
11-Keto-beta-bosw	17019-92	-	C30H46O4	470.33961	471.3	453.3	+	9.23923	172545.9365	5985.05	4002.15	72118.1	37260.8	72571	7254.28	14994.3	9793.12
Alisol A	19885-10	C17459	C30H50O5	490.365825	473.4	383.3	+	7.26725	446.907	937.089	3555.75	4604.41	3589.89	5867.44	7901.91	989.564	4225.94
Alphitolic acid	19533-92	C16912	C30H48O4	472.35526	473.4	409.3	+	10.5567	1845		6430.29	9032.5	2930.42	26191.4	4609.72	2371.41	594.072
Bryodulcosigenin	4965-97-	C08789	C30H50O4	474.37091	475.4	439.4	+	9.93572	1938.246	3346.55	3657.03	6748.47	5275.65	41850.1	37296.2	3363.86	4442.53
Cucurbitacin B	6199-67-	C08794	C32H46O8	558.31927	557.3	497.3	-	11.1853	588.4644997	875.054	9300.4	8268.38	1609.46	326.794	216.918	628.253	1310.77
Cucurbitacin E	18444-66	C08797	C32H44O8	556.30362	557.3	497.3	+	11.9302	4819.999998	806.859	3021.91	2251.5		7079.91	11993.5	17220.3	7607.14
Cycloeucalenol	469-39-6	C02141	C30H50O	426.386165	427.4	83.1	+	11.8993	85643.35749	9675.16	34351.2	73241.9	29471.6	15436.5	263095	46181.2	31136
Erythrodiol;16alph	545-48-2	C20945	C30H50O2	442.38108	443.4	407.4	+	12.102	28063.1735	33947	91768.7	22827.9	46939.4	39672.7	45401	11881.2	30318
Friedelin	559-74-0	C08626	C30H50O	426.386165	427.4	369.4	+	12	12266.061		8516.05	11241.3	4006.47		20209	1670.41	3994.97
Ganoderal A	104700-9	-	C30H44O2	436.33413	437.3	419.3	+	8.0896	2970.975	1944.07	6186.84	15191.4	9700.6	18552.7	4392.54	13774	12732.8
Ganoderic acid F	98665-15	-	C32H42O9	570.282885	571.3	553.3	+	12.9734	70418.5655	12061.7	26544.8	8961.51	7065.5	5232.8	21007.6	10805.3	6594.02
Ganoderic acid H	98665-19	-	C32H44O9	572.298535	573.3	555.3	+	11.7315	10842.16799	13539	19001.2	8198.31	33197.9	56735.2	6800.46	15175.9	13830.7
Ganoderol A	104700-9	-	C30H46O2	438.34978	439.4	421.3	+	11.3119	4205.232505	12587.6	12655.5	15644.5	89951.4	198775	15304.2	14008.5	31976.5
Ganoderol B	104700-9	-	C30H48O2	440.36543	441.4	405.4	+	11.2112	4415.202505	11435.5	6826.12	10671.8	17658.5	13375.4	9366.99	21532	20620.2
Germanicol	465-02-1	C19833	C30H50O	426.386165	427.4	409.4	+	12.631	84429.19603	107091	149734	111654	129445	33057.2	75748.7	87695.9	129717
Glycyrrhetic acid 3	34096-83	-	C36H54O10	646.3717	647.4	453.3	+	14.0272	4092.720501		4121.38	2635.29	3498.04	3034.84	3997.25	4356.26	3067.14
Glycyrrhetinic acid	471-53-4	-	C30H46O4	470.33961	471.3	189.2	+	9.22772	270.165	270.912	1184.59	3953.94	2471.8	21331	831.085	2550.03	2080.82
Jangomolide	93767-25	-	C26H28O8	468.17842	469.2	451.2	+	13.7883	3436.890001	7274.13	8968.86	5398.28	2990.88	2801.09	11635	5608.78	6185.65
Karounidiol	118117-3	C17394	C30H48O2	440.36543	441.4	405.4	+	10.5522	3572.059998	4700.42	2325.42	7546.53	4108.41	33471.4	22313.8	1768	3699
Limonexic acid	99026-99	-	C26H30O10	502.1839	503.2	485.2	+	12.5539	25543.7185	23225.8	38856.5	35075.5	12520.3	32288	14351.7	26506.4	45083.5
Lucidadiol	252351-9	-	C30H48O3	456.360345	457.4	421.3	+	12.642	5138.000003	6144.42	11487.4	8185.57	6003.44	15388.1	4918.35	2475.23	4428.56
Lupeol	545-47-1	C08628	C30H50O	426.386165	427.4	109.1	+	11.9033	48390.82951	130235	35141.9	313075	26592.7	40076.1	3161255	35100.4	25703.3
Rutaevin	33237-37	C08779	C26H30O9	486.188985	487.2	469.2	+	12.5814	30047.5015	3167.23	15714		11520	11006.7	3770	11451.2	20454.8
Shionone	10376-48	C17966	C30H50O	426.386165	427.4	409.4	+	12	36839.64249	105605	77268.6	177004	21131.9	20729.1	535554	102207	50561.5
Simiarenol	1615-94-	C17901	C30H50O	426.386165	427.4	409.4	+	12.6546	199521.433	107091	149734	111654	129445	33057.2	75748.7	87695.9	63289.6
Taraxasterol	1059-14-	C08636	C30H50O	426.386165	427.4	409.4	+	10.3721	1845	6739.43	5335.5	9055.58	3648.42	17376.3	2264.88	3558	2486.71
Tetrahymanol	2130-17-	C06083	C31H54O	442.417465	443.4	425.4	+	10.5735	2930.586	6923.95	3788.54	9293.56	7964.88	14275.9	5119.97	5882.84	5858.51
Dammarenediol II	14351-29	C19829	C30H52O2	444.39673	445.4	409.4	+	12.6171	53446.93301	25588.3	27930.5	45547.8	61260.8	10446.3	11929.6	4661.16	4095.9
22-Dehydroclerost	26315-07	-	C29H46O	410.354865	411.4	93.1	+	12.5874	14033.00251	16785.2	60300	26152	16046.4	11321.8	7621.29	107865	18563.5
6-Hydroxystigmast	36450-01	-	C29H46O2	426.34978	427.4	409.3	+	10.581	6433.064498	3577.31	8753.33	5984.53	6587.31	18311.2	3517	3952.02	2722.58
Bufotaline	471-95-4	C16923	C26H36O6	444.25119	445.3	385.2	+	11.8993	13887.748	6932.41	7923.44		1607.39	4923	20335		9718
Citrostadienol	474-40-8	C11523	C30H50O	426.386165	427.4	409.4	+	12.2853	45907.311	69566.5	199874	69876.3	61355.8	86040.1	40558.5	31060.4	123464
Fucosterol	17605-67	C08817	C29H48O	412.370515	413.4	353.3	+	12.0752	6505.140499	6447.47	6025.36	4099.15	12830.2		7222.69	9522.86	5636.53
Gamabufotalin	465-11-2	C16962	C24H34O5	402.240625	403.2	367.2	+	6.79732	738.17	755.388	735.745	1222.81	1535.99	16106.1	1557.82	4345.53	1602.92
Hyodeoxycholic ac	83-49-8	C15517	C24H40O4	392.29266	391.3	283.3	-	11.4873	3762.494502		2097.52	5775.97	3313.69		3567.63	2190.2	7158.27
Progesterone	57-83-0	C00410	C21H30O2	314.22458	315.2	97.1	+	11.6043	19657.0545	8069.34	22220.2	16216.8	15336.8	11929.8	1377.35	3574.88	
Testosterone	58-22-0	C00535	C19H28O2	288.20893	289.2	271.2	+	5.9835	1029.848	2995.36	826.863	1162.25	433.356	4764.37	5237.61	8425.96	1749.5
Ursodeoxycholic ac	128-13-2	C07880	C24H40O4	392.29266	375.3	357.3	+	11.4727	8261.123997	6779.39	11970.5	6361.5	15158.2	8314.77	13193.5	13461.8	32239.4
17alpha-Estradiol	57-91-0	C02537	C18H24O2	272.17763	273.2	255.2	+	10.9479	13185.5265	9365.98	14509	3807.48	11845	6546.27	16403.7	9725.89	7208.43
17-Hydroxyproges	3168-01-	C01176	C21H30O3	330.219495	331.2	97.1	+	11.7726	72273.44103	74421.2	59332.8	28879.5	34859	11444.3	1372.35	6574.32	5206.79
7-Oxo-beta-sitoster	2034-74-4		C29H48O2	428.36543	429.4	411.4	+	13.5441	63821.82554	33707.3	97695.7	139649	81335.7	13662.4	15769.7	13427.4	10230.5
Allopregnanolone	516-54-1	C13712	C21H34O2	318.25588	319.3	301.3	+	11.5799	24885.072	31843.4	17819.6	12719.5	15905.1	5364.93	10024.6	14883	7958.8
alpha-Spinasterol	481-18-5	C08840	C29H48O	412.370515	395.4	83.1	+	13.3235	256871.6946	265100	518564	478027	364368	110128	169073	99050.6	133707
Androstanediol	25126-76	C07632	C19H32O2	292.24023	293.2	275.2	+	10.5735	2982.307997	5571	14313.3	3528	14294	608.058	8207.89	4784.6	3728.76
Campesterol	474-62-4	C01789	C28H48O	400.370515	383.4	147.1	+	12.1844	24484.86	19305.5	21952.6	16473.6	7995.64	9435.29	28554	18103.8	28985.4
Cholesterol	57-88-5	C00187	C27H46O	386.354865	369.4	147.1	+	11.8597	36241.5705	16390.9	19782.5	16944.6	12451.6	11795.7	14033.7	10229	8133.52
Corticosterone	50-22-6	C02140	C21H30O4	346.21441	347.2	329.2	+	6.11073	2004.89	4698.64	740.184	3810.59	3180.56	8459.02	2424.63	3786.07	2592.3
Dehydroepiandros	53-43-0	C01227	C19H28O2	288.20893	289.2	253.2	+	5.14703	4633.912499	4836		6082.73	2989.76	3574.45	2304.42	1214.61	2286.1
Deoxycorticosteron	64-85-7	C03205	C21H30O3	330.219495	331.2	97.1	+	9.60557	4843.72	781.344	5799.24	5167.59	6565.28	2075.16	1641.44	734.193	1850.25
Dexamethasone	50-02-2	C06945;C15	C22H29FO5	392.199903	393.2	147.1	+	12	10849.165	15543.6	12912.9	8673.92	16876.5	7015.16	8075.49	11927.9	10083.1
Dihydrotestosteron	521-18-6	C03917	C19H30O2	290.22458	291.2	273.2	+	5.08336	3590	593.739	1218.61	1819.5	2401.4	4307.88	3061.45	4824.44	20503.6
Epiandrosterone	481-29-8	C07635	C19H30O2	290.22458	291.2	273.2	+	5.16513	3590	2255.26	1187.93	1792.5	1999.88	4307.88	7125.89	4824.44	9522.44
Ergosterol peroxide	2061-64-	-	C28H44O3	428.329045	429.3	85.1	+	13.4925	36524.565	4373.88	3374.38	5562.58	5348.59	2386.02	4744.58	1619.84	2153.08
Exemestane	107868-3	C08162	C20H24O2	296.17763	297.2	121.1	+	11.614	2715151.62	643700	2754451	2231369	1389898	22433.8	16833.8	13376.7	17754.8
Helveticoside	630-64-8	C08869	C29H42O9	534.282885	535.3	499.3	+	6.49523	364.0125	838.742	2138.79	2181.87	829.26	16734.7	7729.46	4621.26	2195.93
Lathosterol	80-99-9	C01189	C27H46O	386.354865	369.4	95.1	+	11.8025	53506.485	68629.7	94092.9	19704.8	25755.5	19213.4	12568.5	17977.9	20564.3
Levonorgestrel	797-63-7	C08149	C21H28O2	312.20893	313.2	91.1	+	11.2909	159391.1104	15018.3	187505	369793	207308	19317.2	4409.77	1362.01	12964.8
Nandrolone	434-22-0	C07254	C18H26O2	274.19328	275.2	257.2	+	10.5841	51481.69848	54623.5	36928.1	76931	57410.3	43184.8	52850.3	71613	40558.1
Officinalisinin I	57944-18	C08904	C45H76O19	920.498085	921.5	741.4	+	14.014	1453.9635	1994.01	1736.67	4427.46	2311.17	665.361	1145.48	1086.61	695.639
Physalin A	23027-91	-	C28H30O10	526.1839	527.2	509.2	+	12.1007	12134.63	11622.8	16942.1	22983	19328.7	27441.2	6457.64	9834	13335.6
Physalin D	54980-22	-	C28H32O11	544.194465	545.2	527.2	+	12.1007	24303.33499	12906.7	34600.6	18572.1	14450.5	37614	36872.3	11803.7	13276.7
Physalin G	76045-38	-	C28H30O10	526.1839	527.2	509.2	+	12.1679	10192.75	11622.8	16942.1	41202.9	21043.6	27441.2	6457.64	9834	13335.6
Physalin L;Physali	113146-74-0;120849-		C28H32O10	528.19955	529.2	511.2	+	12.386	23337.91452	19183.2	94237.3	55764.3	34362.2	39425.4	21868.4	7714.77	6592.99
Ruscogenin	472-11-7	C08909	C27H42O4	430.30831	431.3	269.2	+	12.805	-	3111.38	7024.74	5516.08	2011.07		1450.21	3367.26	492.718
Stigmasterol	83-48-7	C05442	C29H48O	412.370515	395.4	83.1	+	13.121	20727.22399	265100	518564	478027	364368	110128	169073	99050.6	133707
Taurolithocholate	516-90-5	C02592	C26H45NO5S	483.301845	484.3	109	+	13.779	2153.961001	2830.66	1658.85	1004.61		2045.12	1531.6	-	891.188
Withanolide A	32911-62	-	C28H38O6	470.26684	471.3	453.3	+	12.2686	78354.33995	17700.7	37994	103568	32293.2	12455	17962.5	21205.9	19577.9

Table S3. RNA-Seq data statistics of *S. baumii*.

Sample	Clean paired reads	Raw data (G)	Clean bases(G)	Q20(%)	Q30(%)	GC content(%)	Clean data ratio(%)
Myc1	25565929	8.18	7.35	98.86	95.6	52.85	89.78
Myc2	24585033	8.17	7.07	98.74	95.25	52.41	86.6
Myc3	23749939	7.91	6.7	98.76	95.29	52.51	84.71
Pri1	26543637	8.73	7.74	98.7	95.11	51.62	88.65
Pri2	26056966	8.64	7.59	98.73	95.2	51.71	87.93
Pri3	25497703	8.41	7.44	98.7	95.1	52.2	88.47
Fru1	21320787	7.01	6.29	98.74	95.26	51.81	89.8
Fru2	20986001	6.93	6.19	98.71	95.15	51.76	89.36
Fru3	18889143	6.28	5.57	98.64	94.94	51.87	88.79

Table S4. Terpenoid synthesis genes were annotated in KEGG.

ko_ID	ko_Annotation	Class	Gene_Number	Gene_ID
ko00900	Terpenoid backbone biosynthesis	Metabolism	21	gene-A7U60_g4165; gene-A7U60_g6678; gene-A7U60_g6774; gene-A7U60_g3991; gene-A7U60_g1906; gene-A7U60_g5051; gene-A7U60_g5414; gene-A7U60_g5285; gene-A7U60_g2533; gene-A7U60_g6282; gene-A7U60_g3826; gene-A7U60_g6623; gene-A7U60_g9137; gene-A7U60_g3101; gene-A7U60_g3827; gene-A7U60_g9141; gene-A7U60_g402; gene-A7U60_g6278; gene-A7U60_g2454; gene-A7U60_g4753; gene-A7U60_g7915
ko00904	Diterpenoid biosynthesis	Metabolism	8	gene-A7U60_g2010; gene-A7U60_g3030; gene-A7U60_g285; gene-A7U60_g6428; gene-A7U60_g6496; gene-A7U60_g3505; gene-A7U60_g6424; gene-A7U60_g6423
ko00908	Zeatin biosynthesis	Metabolism	1	gene-A7U60_g4872
ko00909	Sesquiterpenoid and triterpenoid biosynthesis	Metabolism	5	gene-A7U60_g5199; gene-A7U60_g4830; gene-A7U60_g5118; gene-A7U60_g1440; gene-A7U60_g3573
ko00100	Steroid biosynthesis	Metabolism	22	gene-A7U60_g2221; gene-A7U60_g3386; gene-A7U60_g8217; gene-A7U60_g4943; gene-A7U60_g1239; gene-A7U60_g2849; gene-A7U60_g6749; gene-A7U60_g6315; gene-A7U60_g5073; gene-A7U60_g5199; gene-A7U60_g6255; gene-A7U60_g3232; gene-A7U60_g3573; gene-A7U60_g2718; gene-A7U60_g3496; gene-A7U60_g3495; gene-A7U60_g5741; gene-A7U60_g5123; gene-A7U60_g4198; gene-A7U60_g7819; gene-A7U60_g6571; gene-A7U60_g1788

Table S5. Transcript abundance of terpenoid synthesis genes as per the *S. baumii* transcriptome data annotation.

Pathway	Gene name	Kegg entry	Gene bank accession ID	EC.No.	Read in Myc	Read in Pri	Read in Fru	FPKM Myc	FPKM Pri	FPKM Fru
Terpenoid backbone biosynthesis	AACT	K00626	OCB87509.1	2.3.1.9	2077	2707	943.333	181.13	291.09	233.195
	HMGS	K01641	OCB86311.1	2.3.3.10	1803	495.667	2541.67	127.78	43.5867	293.377
	HMGR	K00021	OCB83928.1	1.1.1.34	5469.67	1198.33	6631.33	124.897	32.9667	212.492
	MVK	K00869	OCB84761.1	2.7.1.36	2268.33	3445	2512	82.8033	150.257	102.143
	PMK	K00938	OCB89752.1	2.7.4.2	562	1293.33	375.333	39.2	109.487	152.068
	MVD	K01597	OCB90355.1	4.1.1.33	1958	3035	1588.33	180.317	346.4	163.44
	IDI	K01823	OCB86091.1	5.3.3.2	2755	1045.33	239	435.713	209.61	49.0183
cis-Zeatin	TRIT1	K00791	OCB88085.1	2.5.1.75	871.333	1092	306.667	49.74	73.4367	14.9683
trans-Zeatin	CYP5340A71	K10717	OCB91754.1	-	135.667	234	0.33333	9.61333	19.7033	0.39667
	CYP5340A72	K10717	OCB92139.1	-	397.333	824.333	6.33333	27.5933	67.0833	22.335
Monoterpenoid	GPS	K14066	OCB89018.1	2.5.1.1	746	169	385.667	47.64	13.16	70.8917
Sesquiterpenoid	FPPS	K00787	OCB92206.1	2.5.1.10	629	4960.33	510.667	68.0733	676.567	1223.23
	ABA4	-	OCB90839.1	-	432.667	1220	10093.3	57.4333	201.49	1186.23
Diterpenoid	GGPS	K13789	OCB88125.1	2.5.1.29	65.3333	107	104	8.46	17.1533	66.205
	CYP512CL1	K04122	OCB86527.1	1.14.14.86	201	50.6667	928	13.8833	4.06	58.2633
	CYP512CL2	K04122	OCB86532.1	1.14.14.86	294	309.333	107	12.21	15.61	4.68167
	CYP512CL3	K04122	OCB86528.1	1.14.14.86	59.6667	330.333	11.3333	4.05333	26.61	1.38167
	CYP512CM1	K04123	OCB90766.1	1.14.14.107	73.6667	229.667	11.6667	5.16333	18.4667	10.8367
Triterpenoid	SQS	K00801	OCB89274.1	2.5.1.21	1623.67	1324.67	160.333	117.857	118.877	59.5183
	SE	K00511	OCB87673.1	1.14.14.17	2180	1053.67	580.667	208.76	126.193	67.6717
Steroid	LS	K01852	OCB89993.1	5.4.99.7	816.667	573.667	602.667	27.9767	23.9533	22.66
	LSD1	K05917	OCB87224.1	1.14.14.154 1.14.15.36	660.667	75.3333	83.3333	39.0133	5.40667	44.1067
	LSD2	K05917	OCB90054.1	1.14.14.154 1.14.15.36	784	649.667	719.667	49.22	49.86	306.492
	ERG24	K00222	OCB87985.1	1.3.1.70	952.333	1931	3695	79.39	196.74	272.807
	MESO1	K07750	OCB84697.1	1.14.18.9	1059.67	99.3333	58	122.737	14.65	95.7983
	ERG26	K07748	OCB87751.1	1.1.1.170	451.667	1049.67	1244.33	36.9767	105.927	96.9867
	ERG27	K09827	OCB86242.1	1.1.1.270	452.333	1058.33	280.333	13.6367	37.8133	45.89
	ERG6	K00559	OCB86578.1	2.1.1.41	1748.67	1710.67	371.333	202.547	251.237	158.158
	ERG2	K09829	OCB90543.1	5.-.-.-	845.333	1559.33	745.667	149.507	357.767	240.75
	ERG3	K00227	OCB89410.1	1.14.19.20	1767	1582	2627.67	92.4333	101.717	156.515
	ERG5	K09831	OCB85193.1	1.14.19.41	1885	527.667	596	124.89	44.23	65.39
	ERG4	K00223	OCB87800.1	1.3.1.71	2574	1827.67	451	198.02	168.163	60.22

(1). Assay procedure for ABA and GA4

1. Addition of standard: Set standard wells and sample wells, and add 50 μL of standard with different concentrations to each standard well.
2. Add sample: set up blank wells (the blank control wells do not add samples and enzyme labeling reagents, and the other steps are the same) and the wells for the samples to be tested. Add 40 μL of sample diluent to the well of the sample to be tested on the enzyme-labeled coating plate, and then add 10 μL of the sample to be tested (the final dilution of the sample is 5 times). Add the sample to the bottom of the well of the microtiter plate, try not to touch the wall of the well, and shake gently to mix.
3. Add enzyme: Add 100 μL of enzyme labeling reagent to each well, except for blank wells.
4. Incubation: Cover the plate with sealing film and incubate at 37°C for 60 min.
5. Liquid preparation: Dilute the 20-fold concentrated washing solution with distilled water 20-fold for later use.
6. Washing: Carefully peel off the sealing film, discard the liquid, spin dry, fill each well with washing liquid, let it stand for 30 s, and then discard, repeat 5 times, and pat dry.
7. Color development: first add 50 μL of color developer A to each well, then add 50 μl of color developer B, gently shake and mix, and develop color at 37°C for 15 min in the dark.
8. Termination: Add 50 μL of stop solution to each well to stop the reaction (the blue turns to yellow at this time).
9. Determination: Zero the blank well, and measure the absorbance (OD value) of each well in sequence at 450 nm wavelength. The measurement should be performed within 15 min after adding the stop solution.

(2). Assay procedure for CZ and TZ

1. Add sample: Set up blank wells (the blank control wells do not add samples and enzyme labeling reagents, and the other steps are the same), standard wells, and sample wells to be tested. Accurately add 50 μL of the standard to the standard wells, add 40 μL of the sample diluent to the well of the sample to be tested, and then add 10 μL of the sample to be tested (the final dilution of the sample is 5 times). Add the sample to the bottom of the well of the microtiter plate, try not to touch the wall of the well, and shake gently to mix.
2. Incubation: Cover the plate with sealing film and incubate at 37°C for 30 min.
3. Dosing: Dilute the 30-fold concentrated washing solution with distilled water 30-fold for later use
4. Washing: Carefully peel off the sealing film, discard the liquid, spin dry, fill each well with washing solution, let it stand for 30 s, and then discard, repeat 5 times, and pat dry.
5. Add enzyme: Add 50 μL of enzyme labeling reagent to each well, except for blank wells.
6. Incubation: The operation is the same as 2.
7. Washing: the operation is the same as 4.
8. Color development: First add 50 μL of color developer A to each well, then add 50 μL of color developer B, gently shake and mix, and let the color develop at 37°C for 10 min in the dark.
9. Termination: Add 50 μL of stop solution to each well to stop the reaction (the blue turns to yellow immediately).
10. Determination: Zero the blank well, and measure the absorbance (OD value) of each well in sequence at 450 nm wavelength. The measurement should be performed within 15 min after adding the stop solution.
